# Complete genome sequence of *Pseudomonas stutzeri* strain RCH2 isolated from a Hexavalent Chromium [Cr(VI)] contaminated site

**DOI:** 10.1186/s40793-017-0233-7

**Published:** 2017-02-08

**Authors:** Romy Chakraborty, Hannah Woo, Paramvir Dehal, Robert Walker, Marcin Zemla, Manfred Auer, Lynne A. Goodwin, Alexey Kazakov, Pavel Novichkov, Adam P. Arkin, Terry C. Hazen

**Affiliations:** 1 Lawrence Berkeley National Laboratory, Berkeley, CA USA; 2 University Of Tennessee, Knoxville, TN USA; 3 Department of Energy Joint Genome Institute, Walnut Creek, CA USA

**Keywords:** *Pseudomonas*, Nitrate reduction, Chromium, Hanford 100H

## Abstract

Hexavalent Chromium [Cr(VI)] is a widespread contaminant found in soil, sediment, and ground water in several DOE sites, including Hanford 100 H area. In order to stimulate microbially mediated reduction of Cr(VI) at this site, a poly-lactate hydrogen release compound was injected into the chromium contaminated aquifer. Targeted enrichment of dominant nitrate-reducing bacteria post injection resulted in the isolation of *Pseudomonas stutzeri* strain RCH2. *P. stutzeri* strain RCH2 was isolated using acetate as the electron donor and is a complete denitrifier. Experiments with anaerobic washed cell suspension of strain RCH2 revealed it could reduce Cr(VI) and Fe(III). The genome of strain RCH2 was sequenced using a combination of Illumina and 454 sequencing technologies and contained a circular chromosome of 4.6 Mb and three plasmids. Global genome comparisons of strain RCH2 with six other fully sequenced *P. stutzeri* strains revealed most genomic regions are conserved, however strain RCH2 has an additional 244 genes, some of which are involved in chemotaxis, Flp pilus biogenesis and pyruvate/2-oxogluturate complex formation.

## Introduction

Hexavalent Cr(VI) is a highly toxic and mobile contaminant in the environment. At the DOE site in Hanford, WA, Cr(VI) concentrations reached as high as 50 ppm as a result of nuclear weapon production waste released into the groundwater and soil. In order to reduce Cr(VI) to non-toxic immobilized Cr(III), the bioremediative strategy at the site has been to stimulate indigenous microorganisms [1] by injecting environmentally safe, food quality polylactate ester Hydrogen Release Compound. The slow release electron donor induced biologically mediated reduction of Cr(VI) to Cr(III) by indigenous microorganisms, and as a result, Cr(IV) concentrations were reduced to below 50 ppb in all parts of the Hanford 100 H site [1]. Some group of organisms including *Pseudomonadaceae* were enriched concomitant to decrease in Cr(VI) concentrations after HRC injection, and continued to remain high [1]. *Pseudomonas stutzeri* strain RCH2, was isolated from a monitoring well post injection.

*Pseudomonas* spp. are well-characterized heterotrophs known to degrade several hydrocarbons [2–5], and reduce metals such as Cr(VI) [6–9]. They have commonly been detected in several DOE contaminated sites [10–13] including Uranium contaminated Oakridge Field Research Center [14, 15]. Prolific cultivation of *Pseudomonas* spp. from such unique contaminated environments is imperative in elucidating the metabolic potential, biochemical and physiological characteristics and the genetic determinants of key pathways of this ubiquitous group of bacteria in the environment. The genome sequence of RCH2 allows for detailed examination of this and closely related microbes in response to environmental perturbations at the genetic level, and provides a basis for investigating response, adaptation and evolution in presence of metal contaminants [16].

## Organism features

### Classification and features

Enrichments were initiated in Minimal Fresh Water medium [17] with 10 mM acetate as the sole electron donor and 10 mM nitrate as the electron acceptor. All enrichments were incubated in the dark at 30 °C. Periodic transfers of positive enrichments as identified by microscopy or visual turbidity, were made into fresh media. After 5 such transfers, a pure culture of strain RCH2 was obtained by the agar shake tube method [18, 19]. For routine culturing, strain RCH2 was grown in MFW medium under anaerobic conditions, using either lactate or acetate as electron donor and nitrate as electron acceptor. All culturing was done in sealed serum vials with N_2_:CO_2_ gas (80:20) in the headspace, as the medium contained 30 mM bicarbonate buffer.

For initial genotyping, gDNA was extracted using the MoBio UltraClean Microbial DNA Isolation Kit (MoBio Inc, Carlsbad, CA). PCR amplification was carried out using universal bacterial 16S ribosomal RNA gene (16S rRNA) primers 1492R and 27 F in 50 μl reactions. The small subunit ribosomal RNA gene was sequenced by Sanger sequencing using universal primers 8 F and 1492R [25] at University of California, Berkeley sequencing facility. 16S rRNA sequence analysis places strain RCH2 in the family *Pseudomonadaceae*.

**Fig. 1:**
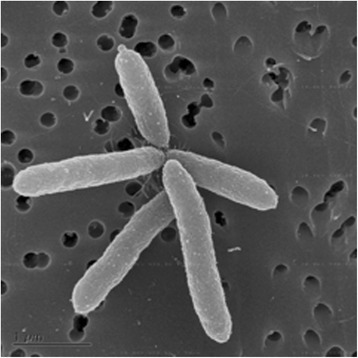
A scanning electron micrograph of *P. stutzeri* strain RCH2 in exponential phase. Scale bar, 1 μm

**Fig. 2:**
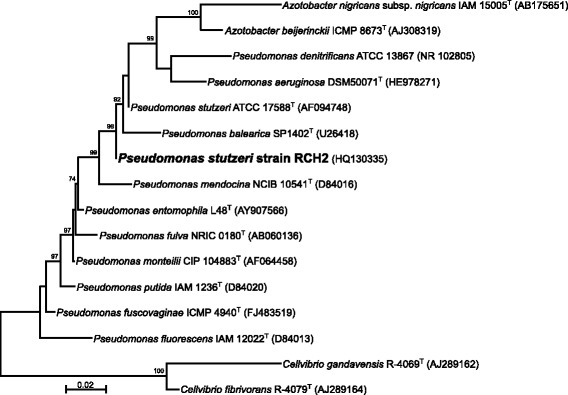
Sequence data were aligned using the Clustal W program [20] with *Pseudomonas* spp. downloaded with strain and accession numbers from the RDP [21] database were sequence identity was 97–100% to *P. stutzeri* strain RCH2. SeaView v4.0 [22] was used to reconstruct the phylogenetic position of *P. stutzeri* strain RCH2 within the genus *Psuedomonas* based on 16S rRNA gene sequence by maximum likelihood following a Tamura-Nei, 93 model and the phylogeny was tested using Approximate Likelihood-Ratio Test (aLRT) (given as a percentage) [23], only values greater than 60% are shown. *Azotobacter* species were included for comparison and *Cellvibrio* species were used for the out-group

**Fig. 3:**
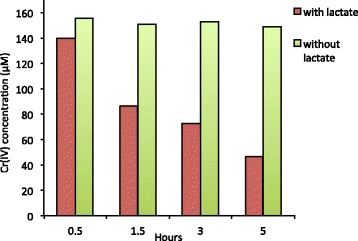
Chromium(VI) reduction by cell suspension of *P. stutzeri* strain RCH2

Cells in exponential phase of strain RCH2 are rod shaped, approximately 2 μm long and 0.25-0.3 μm wide (Figs. 1 and 2). Anaerobically, *P. stutzeri* strain RCH2 grew optimally in MFW medium at 37 °C. While best growth was observed at pH 7.2, strain RCH2 could grow at pH between 6.5–8.0. Growth was observed to decrease with increasing salinity of the medium. Strain RCH2 was tested with and can utilize 10 mM acetate, lactate, fumarate, succinate, pyruvate, glucose and sucrose as electron donors and carbon source while grown under nitrate-reducing conditions. Strain RCH2 can also grow under aerobic conditions as is typical of *Pseudomonas* spp. Strain RCH2 could also grow in complex media such as LB and R2A broth under aerobic conditions. Strain RCH2 reduced Cr(VI) when tested with washed cell suspension. Briefly, strain RCH2 was grown in MFW medium to mid-log phase (optical density of 0.2–0.3 at 600 nm), with lactate as electron donor and nitrate as electron acceptor. Cells were collected by centrifugation, and the cell pellet washed with 30 mM phosphate buffer. Centrifugation and washing were repeated to minimize potential carryover of nitrate in the Cr(VI) reduction experiments. The cell pellet was then resuspended in phosphate buffer and sealed in anaerobic serum vials. To all the vials, 200 μM potassium dichromate was added as electron acceptor, and 10 mM lactate was added as the electron donor. Electron donor addition was left out of the control treatments. The vials were incubated in the dark at 32 °C. Samples were withdrawn periodically for analysis of Cr(VI). Changes in Cr(VI) concentration was determined colorimetrically at 540 nm using the diphenyl carbazide (DPC) assay [24]. The cell suspension experiment demonstrated that after 5 hours, almost 135 μM Cr(VI) was readily reduced by the active cells of strain RCH2 (Fig. 3). In the absence of lactate as the electron donor in the controls, almost no Cr(VI) reduction occurred after 30 min. The reduction of Cr(VI) in the initial period of time could be attributed to abiotic Cr(VI) reduction or carry over lactate from the growth culture despite the washing of the cell pellet.

## Genome sequencing information

### Genome project history

The genome was selected based on the isolate’s ability to thrive in a chromium contaminated aquifer at Hanford 100 H and its ability to reduce toxic Cr(VI). The genome sequence was submitted to NCBI and released on September 6, 2011. Finishing was completed at Los Alamos National Laboratory. A summary of the project information is shown in Tables 1 and 2, which also presents the project information and its association with MIGS version 2.0 compliance.

**Table 1:** Classification and general features of *Pseudomonas stutzeri* strain RCH2 according to the MIGS recommendations [25]

MIGS ID	Property	Term	Evidence code^a^
	Current classification	Domain *Bacteria* Phylum *Proteobacteria* Class *Gammaproteobacteria* Order *Pseudomonadales* Family *Pseudomonadaceae* Genus *Pseudomonas* Species *stutzeri* Strain RCH2	TAS [26] TAS [27] TAS [28] TAS [29, 30] TAS [29, 31] TAS [29, 32, 33]
	Gram stain	Negative	NAS
	Cell shape	Rod-shaped	IDA
	Motility	Motile	IDA
	Sporulation	Non-sporulating	IDA
	Temperature range	Mesophile	IDA
	Optimum temperature	37 °C	IDA
	Carbon source pH range Optimum pH	Lactate, Pyruvate, 6.5–8.0 7.2	IDA
	Terminal electron receptor	Nitrate, Oxygen,	IDA
MIGS-6	Habitat	Cr(VI) contaminated aquifer	IDA
MIGS-6.3	Salinity	Optimal growth at 0.35% salinity	IDA
MIGS-22	Oxygen	Facultative anaerobe	IDA
MIGS-15	Biotic relationship	Free-living	IDA
MIGS-14	Pathogenicity	Unknown	IDA
MIGS-4	Geographic location	Benton County, Washington	IDA
MIGS-5	Sample collection time	2005	IDA
MIGS-4.1	Latitude	Centered on 46°38′51″N	IDA
MIGS-4.2	Longitude	119°35′55″W/46.6475°N 119.59861°W	IDA
MIGS-4.3	Depth		Not reported
MIGS-4.4	Altitude	115.8 m	IDA

^a^Evidence codes - IDA: Inferred from Direct Assay; TAS: Traceable Author Statement (i.e., a direct report exists in the literature); NAS: Non-traceable Author Statement (i.e., not directly observed for the living, isolated sample, but based on a generally accepted property for the species, or anecdotal evidence). These evidence codes are from the Gene Ontology project [34]

**Table 2:** Genome sequencing project information for *Pseudomonas stutzeri* strain RCH2

MIGS ID	Property	Term
MIGS 31	Finishing quality	Finished
MIGS-28	Libraries used	454 titanium standard library, 454 paired end library, Illumina GAii shotgun library
MIGS 29	Sequencing platforms	454-GS-FLX, Illumina GAii
MIGS 31.2	Fold coverage	454: 32.2x Illumina GAii: 127.1x
MIGS 30	Assemblers	Newbler, Velvet
MIGS 32	Gene calling method	GenePrimp, Prodigal 1.4
	Locus Tag	PSEST
	Genbank ID	CP003071.1- CP003074.1
	Genbank Date of Release	September 6, 2011
	GOLD ID	Gp0005131
	BIOPROJECT	PRJNA60029
MIGS 13	Source Material Identifier	
	Project relevance	Chromium (VI) reduction, nitrate reduction

### Growth conditions and genomic DNA preparation

*P. stutzeri* strain RCH2 was grown under anaerobic conditions at 37 °C in basal medium containing 20 mM lactate as the sole electron donor and carbon source and 10 mM nitrate as the terminal electron acceptor. Cells were harvested for DNA extraction when they reached mid-log phase of growth.

Genomic DNA was extracted from a 50 ml culture using the CTAB extraction method recommended by JGI, USA [35]. JGI DNA mass standards were used to ascertain the quantity and quality of the extracted gDNA. JGI protocol for running the gel electrophoresis was followed.

### Genome sequencing and assembly

The genome of *P. stutzeri* strain RCH2 was generated at the DOE JGI using a combination of Illumina [36] and 454 technologies [37]. For this genome we constructed and sequenced an Illumina GAii shotgun library which generated 16,378,443 reads totaling 589.6 Mb, a 454 Titanium standard library which generated 255,080 reads and 2 paired end 454 libraries with an average insert size of 9 kb, and 19 kb which generated 582,773 reads totaling 216.3 Mb of 454 data. All general aspects of library construction and sequencing performed at the JGI [35]. The initial draft assembly contained 32 contigs in 1 scaffold. The 454 Titanium standard data and the 454 paired end data were assembled together with Newbler, version 2.3. The Newbler consensus sequences were computationally shredded into 2 kb overlapping fake reads (shreds). Illumina sequencing data were assembled with VELVET, version 1.0.13 [38], and the consensus sequence were computationally shredded into 1.5 kb overlapping fake reads (shreds). We integrated the 454 Newbler consensus shreds, the Illumina VELVET consensus shreds and the read pairs in the 454 paired end library using parallel phrap, version SPS −4.24 (High Performance Software, LLC). The software Consed [39–41] was used in the following finishing process. Illumina data were used to correct potential base errors and increase consensus quality using the software Polisher developed at Joint Genome Institute (JGI) (Alla Lapidus, unpublished). Possible mis-assemblies were corrected using gap Resolution (Cliff Han, unpublished), Dupfinisher [42], or sequencing cloned bridging PCR fragments with subcloning. Gaps between contigs were closed by editing in Consed, by PCR and by Bubble PCR (J-F Cheng, unpublished) primer walks. A total of 68 additional reactions were necessary to close gaps and to raise the quality of the finished sequence. The total size of the genome is 4,600,489 bp and the final assembly is based on 148 Mb of 454 draft data which provides an average 32.2x coverage of the genome and 584.6 Mb of Illumina draft data which provides an average 127.1x coverage of the genome.

### Genome annotation

Genes were identified using Prodigal [43] as part of the Oak Ridge National Laboratory genome annotation pipeline, followed by a round of manual curation using the JGI GenePRIMP pipeline [44]. The predicted CDSs were translated and used to search the National Center for Biotechnology Information (NCBI) nonredundant database, UniProt, TIGRFam, Pfam, PRIAM, KEGG, COG, and InterPro databases. These data sources were combined to assert a product description for each predicted protein. Non-coding genes and miscellaneous features were predicted using tRNAscan-SE [45], RNAMMer [46], Rfam [47], TMHMM [48], and signalP [49].

## Genome properties

**Fig. 4:**
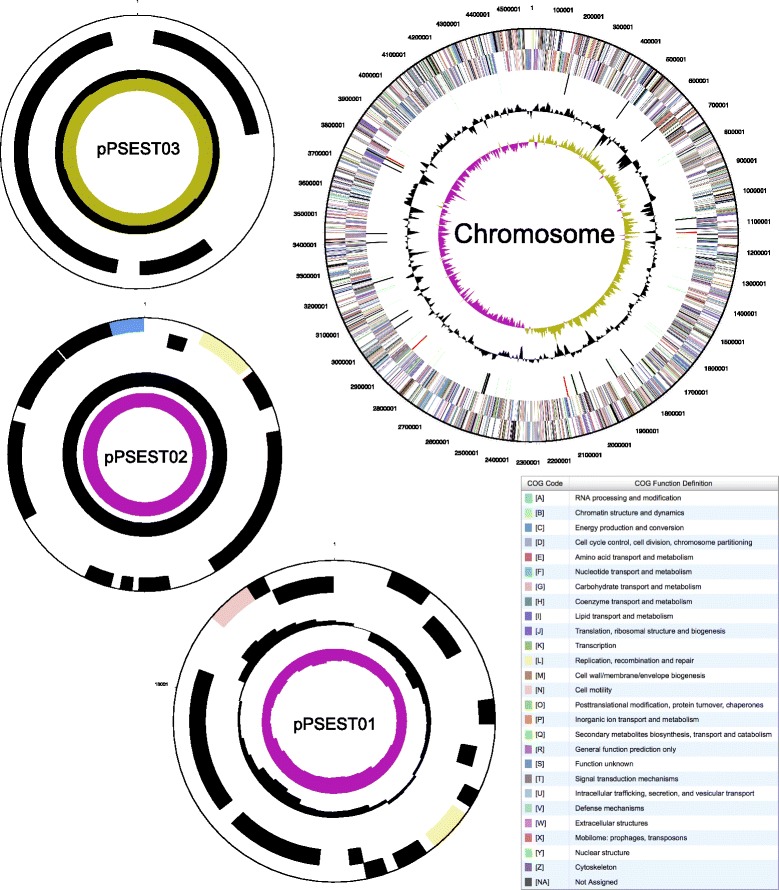
Graphical map of the chromosome and plasmids of *P. stuzeri* strain RCH2. From outside to center of each map: Genes on forward strand (color by COG categories as denoted by the JGI Integrated Microbial Genomes (IMG) platform), Genes on reverse strand (color by COG categories), RNA genes (tRNAs green, sRNAs red, other RNAs black), GC content, GC skew

The genome consists of one circular chromosome of 4,575,057 bp (62.49% GC content) and includes 3 circular plasmids of 12,763 bp, 9,865 bp and 2,804 bp for a total genome size of 4,600,489 bp. There are 4322 protein-coding genes of which 3593 genes were assigned to a putative function and the 729 remaining genes were annotated as hypothetical proteins. The properties and the statistics of the genome are summarized in Tables 3, 4 and 5 and Fig. 4.

**Table 3:** Summary of genome: 1 chromosome and 3 plasmids

Label	Size (Mb)	Topology	INSDC identifier	RefSeq ID
Chromosome	4.575	circular	CP003071.1	NC_019936.1
Plasmid pPSEST01	0.013	circular	CP003072.1	NC_019937.1
Plasmid pPSEST02	0.010	circular	CP003073.1	NC_019938.1
Plasmid pPSEST03	0.003	circular	CP003074.1	NC_019939.1

**Table 4:** Genome statistics for *Pseudomonas stutzeri* strain RCH2

Attribute	Value	% of Total
Genome size (bp)	4,600,489	100.00
DNA coding (bp)	4,159,553	90.42
DNA G + C (bp)	2,874,963	62.49^a^
DNA scaffolds	4	100.00
Total genes	4,412	100.00
Protein coding genes	4,322	97.96
RNA genes	90	2.04
Pseudo genes	57	1.29^b^
Genes in internal clusters	NA	
Genes with function prediction	3,593	81.44
Genes assigned to COGs	3,195	72.42
Genes with Pfam domains	3,786	85.81
Genes with signal peptides	477	10.81
Genes with transmembrane helices	1,118	25.34
CRISPR repeats	NA	

^a^GC percentage shown as count of G's and C's divided by the total number of bases. The total number of bases is not necessarily synonymous with a total number of G's, C's, A's, and T's

^b^Pseudogenes may also be counted as protein coding or RNA genes, so is not additive under total gene count

**Table 5:** Number of genes associated with the general COG functional categories

Code	Value	% of total^a^	Description
J	227	6.27	Translation, ribosomal structure and biogenesis
A	1	0.03	RNA processing and modification
K	237	6.55	Transcription
L	129	3.56	Replication, recombination and repair
B	1	0.03	Chromatin structure and dynamics
D	39	1.08	Cell cycle control, Cell division, chromosome partitioning
V	83	2.29	Defense mechanisms
T	243	6.71	Signal transduction mechanisms
M	216	5.97	Cell wall/membrane biogenesis
N	158	4.37	Cell motility
U	78	2.16	Intracellular trafficking and secretion
O	155	4.28	Posttranslational modification, protein turnover, chaperones
C	251	6.94	Energy production and conversion
G	169	4.67	Carbohydrate transport and metabolism
E	285	7.88	Amino acid transport and metabolism
F	85	2.35	Nucleotide transport and metabolism
H	171	4.73	Coenzyme transport and metabolism
I	172	4.75	Lipid transport and metabolism
P	241	6.66	Inorganic ion transport and metabolism
Q	91	2.51	Secondary metabolites biosynthesis, transport and catabolism
R	302	8.34	General function prediction only
S	230	6.36	Function unknown
-	1217	27.58	Not in COGs

^a^The total is based on the total number of protein coding genes in the annotated genome

## Insights from the genome sequence

**Fig. 5:**
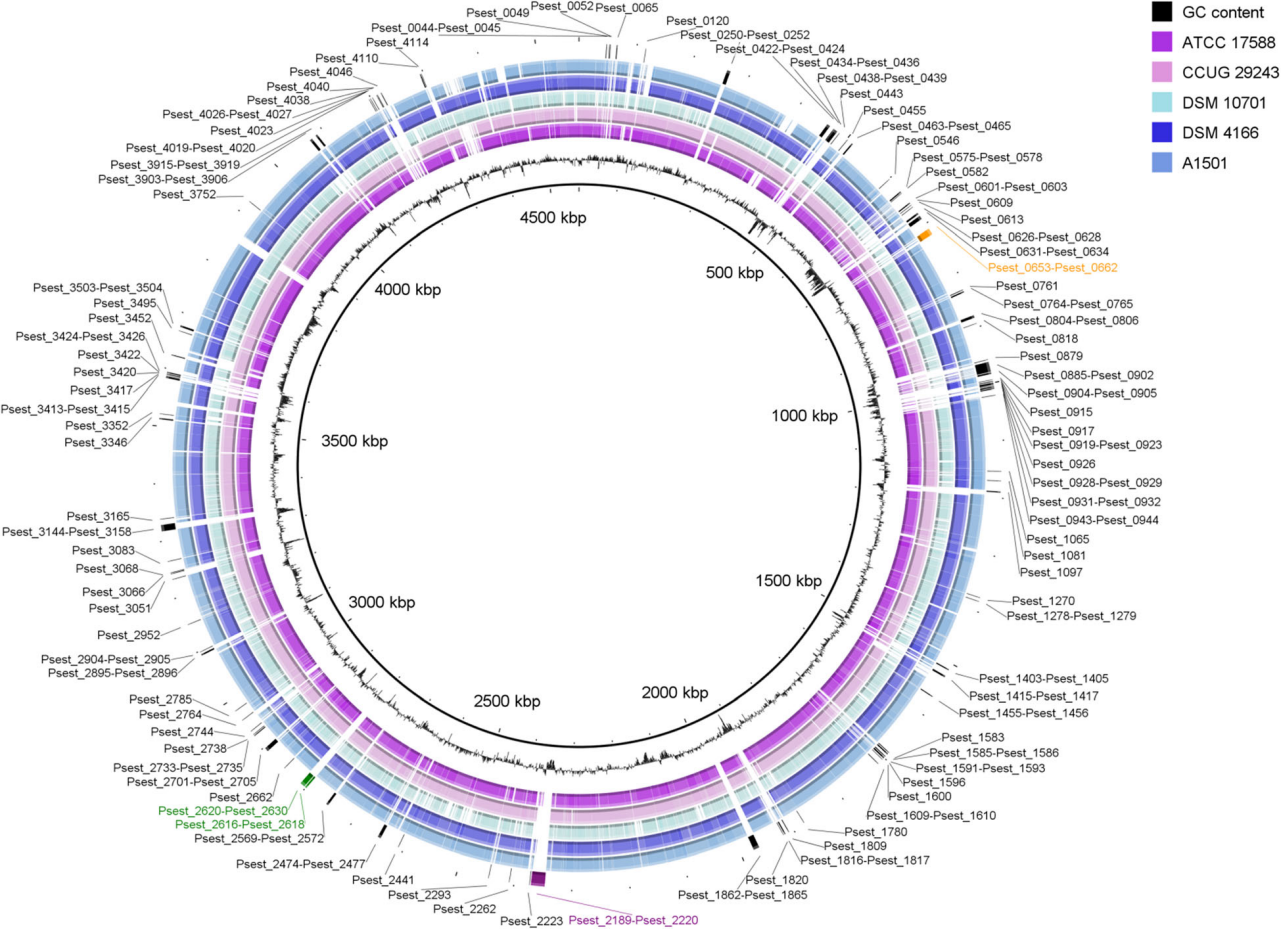
Global comparison of six *P. stutzeri* strains with reference to the RCH2 strain chromosome sequence. We aligned each of the individual genome sequences against the RCH2 chromosome sequence using Basic Local Alignment Search Tool BLASTN [51]. The innermost ring indicates the genomic position. The next ring is a plot of G + C content. Next five rings indicate the presence or absence of BLAST hits in that position, with each ring corresponding to one of *P. stutzeri* strains. The outermost ring indicates positions of RCH2-specific genes, with clusters of chemotaxis, pyruvate dehydrogenase and Flp pili genes marked orange, purple and green, respectively. The graphical view of the alignments was rendered using BLAST Ring Image Generator [52]

Global genomic comparison of six fully sequenced *P. stutzeri* strains (RCH2, A1501, ATCC 17588, CCUG 29243, DSM 4166, DSM 10701) demonstrated that most of the genomic regions are conserved but there are some differences between genome of RCH2 and other genomes (Fig. 5). We identified genes that are differentially present in RCH2 and other fully sequenced *P. stutzeri* strains by using “Compare two proteomes” tool of DOE Systems Biology Knowledgebase [www.kbase.us]. For 4231 proteins encoded by chromosomal genes of RCH2 strain, there are 3696, 3677, 3534, 3526 and 3199 orthologous genes in CCUG 29243, DSM 4166, A1501, ATCC 17588 and DSM 10701 strains, respectively. No orthologs for plasmid genes of RCH2 were found in five other *P. stutzeri* strains. We identified 244 *P. stutzeri* genes that are present in RCH2 chromosome but absent in all other fully sequenced strains. Approximately 48% of those genes encode hypothetical proteins. Particularly noticeable are RCH2-specific gene clusters encoding chemotaxis (Psest_0653-Psest_0662), pyruvate/2-oxoglutarate complex (Psest_2217-Psest_2220) and Flp pilus biogenesis (Psest_2616-Psest_2630) proteins (Fig. 5). We identified 18 strain-specific genes encoding transcriptional regulators, thus the regulatory network of *P. stutzeri* RCH2 may differ significantly from closely related bacteria.

## Extended insights

We searched for regulatory interactions in *P. stutzeri* strain RCH2 using an automated conservative propagation procedure described earlier [50]. By comparison with the RegPrecise database, this procedure identified 27 regulons in *P. stutzeri* RCH2 genome. Of those regulons, 11 contain genes for central carbon metabolism and utilization of various carbon sources. Other regulatory systems control metabolism of amino acids (MetR, PhhR), nitrogen (NtrC) and phosphonate (PhnF), biosynthesis of biotin (BirA), lipopolysaccharide (GlmR) and nucleotides (NrdR, RutR), metal homeostasis (CadR, CueR, Zur), DNA repair (LexA) and biogenesis of iron-sulfur clusters (IscR). At the same time, *P. stutzeri* strain RCH2 lacks several transcription factors conserved in various *Gammaproteobacteria*, like PdxR (regulator of pyridoxine biosynthesis), FabR (regulator of fatty acid biosynthesis) and SoxR (regulator of superoxide stress response).

## Conclusion

*Pseudomonas stutzeri* strain RCH2 isolated from chromium-contaminated aquifer, is a complete denitrifier that can couple nitrate reduction to oxidation of several organic carbon. When supplemented with lactate, robust culture of strain RCH2 reduces Cr(VI) rapidly and this feature contributes to the versatility of this organism to survive in such chromium(VI) contaminated areas. The genome of strain RCH2 reveals differences when compared to closely related strains, and contains an additional 244 genes, mostly of unknown function. Clusters that are specific to strain RCH2 include chemotaxis and Flp pilus biogenesis and these clusters are absent from the five closely related strains examined. The genome sequence of strain RCH2 will assist in further research into the underlying mechanisms of adaption and persistence in metal and/or nitrate contaminated sites.

## Abbreviations

DOE: Department of Energy
gDNA: Genomic DNA
JGI: Joint Genome Institute
NCBI: National Center for Biotechnology Information (Bethesda, MD, USA)
RDP: Ribosomal Database Project (East Lansing, MI, USA)
